# Dual contrast CMR for evaluation of telmisartan and amlodipine combination therapy in the diabetic murine myocardial injury model

**DOI:** 10.1186/1532-429X-15-S1-P34

**Published:** 2013-01-30

**Authors:** Paul J Kim, Yongquan Gong, Xiaohu Ge, Rajesh Dash, Ildiko Toma, Phillip Harnish, Robert C Robbins, Phillip C Yang

**Affiliations:** 1 Medicine, Stanford, Menlo Park, CA, USA; 2 Eagle Vision Pharmaceutical Corporation, Downington, PA, USA; 3 Dept of Cardiothoracic Surgery, Stanford University Medical Center, Stanford, CA, USA; 4 Advanced Bionics, Valencia, CA, USA

## Background

Combination therapy has been shown to improve cardiovascular outcomes in hypertensive, diabetic patients. Amlodipine demonstrates beneficial pleiotropic effects including improved endothelial function, enhanced angiogenesis, and reduced cardiovascular risk. Telmisartan exhibits similar beneficial cardiovascular effects in diabetic patients. Amlodipine in combination with telmisartan may provide a synergistic benefit. In this study, dual contrast CMR, using manganese (manganese-enhanced MRI; MEMRI) and gadolinium (delayed-enhanced MRI; DEMRI), will evaluate the viable myocardium and myocardial scar, respectively, to investigate the underlying mechanism of combined telmisartan and amlodipine therapy.

## Methods

Acute myocardial injury (AMI) was created by ligation of the left anterior descending coronary artery (LAD) in db/db mice. The mice were allocated to two groups: (1) tap water (n = 17) and (2) addition of telmisartan (10 mg/kg/day) and amlodipine (10 mg/kg/day) in the tap water per os ad libitum following 1 week of convalescence after AMI (n = 23). Dual contrast CMR with manganese (MEMRI) and gadolinium (DEMRI) was performed weekly with treatment to obtain LVEF and assess viable myocardium and scar myocardium. After the 4-week follow up period, contractile function was assessed by pressure-volume (PV) loop analysis. Flow cytometry of venous blood was performed to determine the percentage of circulating endothelial progenitor cells. Real-time PCR of the explanted myocardium was performed to evaluate the expression of collagen I and III, CTGF, TGFβ, fibronectin and Akt.

## Results

We demonstrate significantly improved left ventricular function at weeks 1, 2, and 4 following combined telmisartan and amlodipine therapy (32.0% ± 4.8%*, 31.8% ± 3.9%*, and 35.3% ± 2.3%*) compared to control (18.9% ± 1.5%, 21.8% ± 1.3%, and 15.7% ± 3.4%, *p < .05). PV loop analysis also demonstrates improved systolic and diastolic parameters with significantly increased maximum pressure and dp/dt max and significantly decreased LV end-diastolic pressure and Tau. MEMRI demonstrates significantly increased viable myocardial volumes in the treated mice (87.2% ± 0.92%*, 90.2% ± 1.3%*, and 91.4% ± 1.1%*) compared to control (80.5% ± 7.6%, 79.9% ± 3.1%, and 79.9% ± 3.1%, *p<0.05) throughout the study. DEMRI scar volumes initially show no difference but demonstrate a significantly decreased scar volume after 4 weeks of treatment. Furthermore, the measurement of endothelial progenitor cells demonstrate a dramatic increase in the treated compared to control mice (98.1% ± 0.25%* vs. 5.0% ± 2.6%, *p<0.05). However, no differential expression of fibrotic genes by PCR was observed.

## Conclusions

This study provides potential evidence of salvage of the injured myocardium by combined telmisartan and amlodipine therapy, which could underlie the mechanism of myocardial restoration after ischemic injury. Dual contrast CMR accurately identifies the dynamic effects on both the viable myocardium and myocardial scar.

## Funding

T32 CVIS Grant under National Institute of Biomedical Imaging and Bioengineering

